# *Eumorphusmarginatus* group in Sulawesi, Indonesia (Coleoptera, Endomychidae)

**DOI:** 10.3897/zookeys.820.31527

**Published:** 2019-01-30

**Authors:** Hiroyuki Yoshitomi, Koichi Sogoh

**Affiliations:** 1 Entomological Laboratory, Faculty of Agriculture, Ehime University, Tarumi 3-5-7, Matsuyama, 790-8566, Japan Ehime University Matsuyama Japan

**Keywords:** Coccinelloidea, Lycoperdininae, new species, Oriental Region, taxonomy

## Abstract

The species of the *Eumorphusmarginatus* group from Sulawesi are revised. Two previously known species, *E.costatus* and *E.wegneri*, are redescribed, and a new species, *Eumorphusmirabilis***sp. n.**, is described. A key for identification of these species is provided.

## Introduction

*Eumorphus* Weber, 1801 is the largest genus in the subfamily Lycoperdininae of the family Endomychidae. It includes 77 species/subspecies (Shockley et al. 2009, Chang and Ren 2017). Strohecker (1968) classified species of this genus into seven species groups (three species were undetermined). Of these, the *Eumorphusmarginatus* group (sensu Strohecker 1968) includes well-known beetles having a unique body shape like tortoise beetles (Chrysomelidae, Hispinae). Approximately 30 species (including subspecies) are known in this species group (Strohecker 1968), but the knowledge about most species is limited and reexamination is needed. For example, *Eumorphusmarginatus* Fabricius, 1801, the largest and most well-known species (Arrow 1925), has been recorded from Borneo, Java, Sumatra, Papua New Guinea, the Philippines, Myanmar, and Lanyu (Kano 1928). This species seems to be widely distributed, but its distribution and taxonomic status needs revision. In addition, the endophallic structure of the subfamily Lycoperdininae is diverse in shape, size, and construction (e.g., Tomaszewska 2005), but this has been scarcely studied so far.

In the present paper, we describe a remarkable new species from Sulawesi, Indonesia, and provide redescriptions of two previously known species of *E.marginatus* group from this island. The male genitalia of these species including endophallic structure are also described.

## Materials and methods

The material examined in this paper is lodged in Ehime University Museum, Matsuyama, Japan (**EUMJ**) and National Museum of Nature & Science, Tsukuba, Japan (**NSMT**). General observations, dissections, and microstructures of the dissected parts were made under a Leica MZ95. After observation, the dissected parts were mounted on the same card with the specimen. Photographs were taken under the Leica MZ95.

Morphological abbreviations used in this study are as follows:

**EL** elytral length from anterior margin to elytral apex, along suture including scutellum;

**EW** maximum elytral width;

**PLM** pronotal length in median line;

**PLS** pronotal length from anterior angle to posterior margin;

**PWA** pronotal width in anterior angles;

**PWP** pronotal width in posterior angles;

**TL** total length (PLM + EL).

The average measurement is given in parentheses after the range. Technical terms follow Tomaszewska (2005) and Sogoh and Yoshitomi (2017). The orientations of the aedeagus in the abdomen are as follows: the view with the apical and subapical branches situated on the right side is ventral (Fig. 6A, E, I), upside is right lateral (Fig. 6B, F, J), and left side is dorsal (Fig. 6C, G, K). The label data of the specimen examined is cited verbatim in the original spelling and given in quotation marks (“…”).

## Taxonomy

### Eumorphus

Taxon classificationAnimaliaColeopteraEndomychidae

Weber, 1801

#### Type species.

*Eumorphussumatrae* Weber, 1801 (= *Erotylusquadriguttatus* Illiger, 1800).

#### Diagnosis.

*Eumorphus* is most similar to *Platindalmus* and *Gerstaeckerus*, but is distinguished from these by the following combination of characters: 1) lateral margin of pronotum with a tendency to form irregularly broken lines, inconsistent and often asymmetrical; 2) apex of mandibles narrowly chisel-shaped; 3) basal margin of elytra simple; 4) intercoxal process of mesoventrite subparallel-sided; 5) male femora lacking fringes of long hairs on inner edges (after Tomaszewska 2005).

The *Eumorphusmarginatus* group (sensu Strohecker 1968) is characterized as follows: large body size; elytra with lateral flattened margins wide, elevated dorsally in mesal part in male; male fore tibiae with sexual dimorphism.

#### Biological notes.

*Eumorphusmarginatus* Fabricius, 1801 was collected from *Polyporus* sp., and living individuals apparently have a strong unpleasant smell, much like that of the seeds of *Parkiaspeciosa* (after Arrow 1925). The larva of *Eumorphusquadriguttatus* (Illiger) was described by Bugnion (1909) and Hayashi (1986), and Tomaszewska (2005) provided a description of the larva of a *Eumorphus* sp. with figures.

##### Key to the species of *Eumorphusmarginatus* group in Sulawesi

**Table:** 

1	Elytra with two anterior spots	***E.mirabilis* sp. n.**
–	Elytra with two anterior and two posterior spots	**2**
2	Elytra cordiform; elytral spots small, distant from suture by 2 times spot’s diameter	*** E. wegneri ***
–	Elytra bluntly rounded behind; elytral spots relatively large, each distant from suture by its own diameter	*** E. costatus ***

### Eumorphus costatus

Taxon classificationAnimaliaColeopteraEndomychidae

Gorham, 1873

**Figure 1.**
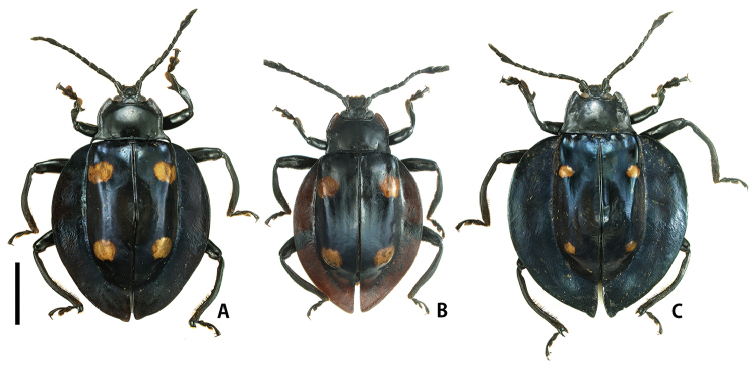
Habitus of *Eumorphus* spp. **A***E.costatus*, male **B***E.costatus*, female **C***E.wegneri*, male. Scale bar: 5.0 mm.

Figs 1A, B ; 3A, B ; 4A, B ; 5A, B ; 6A–D

Eumorphus costatus Gorham, 1873: 34, fig. 6. Strohecker 1968: 84, figs. 11, 65 (short description and figures of habitus and male genitalia).

#### Material examined.

1 male and 2 females (EUMJ), “C. Sulawesi: Puncak Palopo Puncak, Indonesia, 23–25.XI.2012, Kiyoshi Ando leg.”; 1 male (EUMJ), “(C. Sulawesi) Dongidongi, 10 km S of Palolo, alt. ca 1000 m, 18.VI.1986, S. Nagai leg.”; 2 female (EUMJ), “INDONESIA Puncak Palopo Sulawesi Selatan 30–31.XII.1999 N. Ohbayashi leg.”; 1 male & 1 female, “[INDONESIA] Bantu Kaya Angin Battang Palopo Sulawesi Selatan Alt. ca. 350 m 02°57'S, 120°08'E 31.I.2013 J. Yamasako leg.”; 1 female (EUMJ), “[BONEBOLANGO: INDONESIA] Mt. Tilongkabila (Gunung Tilonkabila) N. Sulawesi, alt. ca. 1300–1500 m 0°35'18.14"N, 123°13'22.71"E–0°35'18.37"N, 123°13'22.61"E 10.VI.2012 Ryo Ogawa leg.”

#### Diagnosis.

This species is similar to *Eumorphusmarginatus* Fabricius, 1801 but differs from the latter in the following characteristics: elytral spots smaller, each distant from suture by its own diameter; in *E.marginatus* elytral spots larger and distant from suture by 1/2 of its own diameter).

#### Description.

*Male*.

Coloration of body black, with blueish luster; elytral spots yellowish orange.

Antennae long, reaching to ca. basal 1/4 of elytra.

Pronotum (Fig. 4A) glossy, narrowly depressed in lateral parts; lateral margins subparallel-sided in basal 1/2, then gently tapered anteriorly; lateral sulci relatively long, reaching ca. basal 1/3 of PLM; anterior angles ca. 60°; posterior angles sharply pointed, projecting postero-laterally; PLM/PLS 0.85–0.86 (0.86); PWP/PWA 1.84–1.95 (1.89); PWP/PLM 1.79–1.99 (1.89); PWP/PLS 1.55–1.69 (1.62). Elytra (Fig. 1A) ovate, bluntly rounded behind, widest at middle; lateral flattened margins wide, flat dorsally; elytral spots two pairs, relatively large, each distant from suture by its own diameter; anterior ones transverse, situated in anterior 1/4 of elytra; posterior ones oval, situated in posterior 1/3 of elytra; elytra along suture (Fig. 3A) distinctly elevated in mesal part; EL/EW 1.06–1.09 (1.07); EL/PLM 4.09–4.40 (4.24); EW/PWP 2.04–2.14 (2.09); TL/EW 1.32–1.33 (1.33). Fore tibiae (Fig. 5A) stout; apical carinae distinct, covered with yellowish brown pubescence on inner surface; fore tibial tooth long, triangular, situated in apical 1/4. Metatibial apices widely prolonged and flattened, sharply pointed at outer angles.

Aedeagus (Fig. 6A–D) 5.4 mm long; median lobe stout, expanded ventrally in median part; apical branch short, obtuse and bisinuate at apex; subapical branch long, evenly curved apically; tegmen not movable, with short tegminal strut; endophallus consisting of bifid long right membranous lobe and left membranous lobe, with rather sclerotized spinous plate at base of left lobe.

*Female*. Similar to male, but sexual dimorphism distinct in the following characteristics: posterior angles of pronotum (Fig. 4B) nearly right-angled; elytra with lateral flattened margins narrower than in male, gently arcuate dorsally along suture (Fig. 3B); fore tibiae (Fig. 5B) slender and straight, covered with yellowish brown pubescence in apical half; metatibial apices simple; PLM/PLS 0.80–0.84 (0.82); PWP/PWA 1.46–1.69 (1.59); PWP/PLM 1.59–1.77 (1.66); PWP/PLS 1.31–1.44 (1.37); EL/EW 1.10–1.32 (1.24); EL/PLM 3.86–4.25 (4.07); EW/PWP 1.93–2.13 (1.98); TL/EW 1.37–1.63 (1.55).

*Measurements*. Male (n = 2). TL 16.68–18.53 (17.61) mm; PW 5.88–6.83 (6.36) mm; PW 3.20–3.50 (3.35) mm; PL 3.28–3.43 (3.36) mm; PL 3.80–4.05 (3.93) mm; EL 13.40–15.10 (14.25) mm; EW 12.6–13.9 (13.25) mm. Female (n = 6). TL 13.40–16.85 (15.17) mm; PW 4.55–5.60 (4.96) mm; PW 2.90–3.35 (3.12) mm; PL 2.60–3.30 (2.99) mm; PL 3.20–4.00 (3.63) mm; EL 10.80–13.55 (12.18) mm; EW 8.85–10.85 (9.79) mm.

#### Distribution.

Indonesia (Sulawesi: South, Central, and North).

### Eumorphus wegneri

Taxon classificationAnimaliaColeopteraEndomychidae

Strohecker, 1956

Figs 1C ; 3C ; 4C ; 5C ; 6E–H

Eumorphus wegneri Strohecker, 1956: 245; Strohecker 1968: 84, figs. 10, 64 (short description and figures of habitus and male genitalia).

#### Material examined.

1 Male (EUMJ), “W. Sulawesi: Mamasa Kampung Busau, Sulbar, Indonesia, 21.XI.2012, Kiyoshi Ando leg.”

#### Diagnosis.

This species is distinct in having cordiform elytra and small elytral spots distant from suture.

#### Description.

*Male*.

Coloration of body black, with blueish luster; elytral spots yellowish orange.

Antennae long, reaching ca. basal 1/4 of elytra; antennal club wide.

Pronotum (Fig. 4C) glossy, narrowly depressed/concave in lateral parts; lateral margins gently and tapered anteriorly; lateral sulci short, reaching ca. basal 1/4 of PLM; anterior angles ca. 60°; posterior angles sharply pointed, projecting postero-laterally; PLM/PLS 0.83; PWP/PWA 2.00; PWP/PLM 2.00; PWP/PLS 1.66. Elytra (Fig. 1C) cordiform, widest at middle; lateral flattened margins wide; elytral spots two pairs, small and oval, each distant from suture twice its diameter; anterior ones situated in anterior 1/4 of elytra; posterior ones situated in posterior 1/4 of elytra; elytra along suture (Fig. 3C) distinctly elevated in mesal part; EL/EW 1.03; EL/PLM 4.62; EW/PWP 2.25; TL/EW 1.25. Fore tibiae (Fig. 5C) stout; apical carinae distinct, covered with yellowish brown pubescence in inner surface; fore tibial tooth long and stout, situated in apical 1/4. Metatibial apices prolonged in form of bifid projection; outer angles longer than inner ones.

Aedeagus (Fig. 6E–H) 5.2 mm long, relatively slender; median lobe gently curved ventrally, slightly expanded ventrally in median part; apical branch short, pointed at apex; subapical branch long, winding, curved apically; tegmen movable, with short tegminal strut; endophallus consisting of bifid long right membranous lobe and long left membranous lobe, with rather sclerotized thumb-like projection in dorsal part.

*Female*. Not available for examination.

*Measurements*. Male (n = 1). TL 19.10 mm; PW 6.80 mm; PW 3.40 mm; PL 3.40 mm; PL 4.10 mm; EL 15.70 mm; EW 15.30 mm.

#### Distribution.

Indonesia (Sulawesi: south and west).

### Eumorphus mirabilis sp. n.

Taxon classificationAnimaliaColeopteraEndomychidae

http://zoobank.org/82BE8F98-3293-4D4D-966C-5DA213E52B60

**Figure 2.**
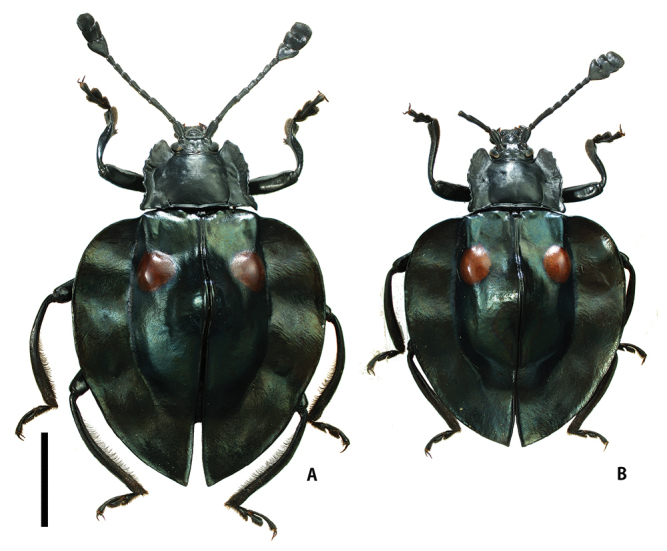
Habitus of *Eumorphusmirabilis* sp. n. **A** Holotype, male **B** paratype, female. Scale bar: 5.0 mm.

**Figure 3.**
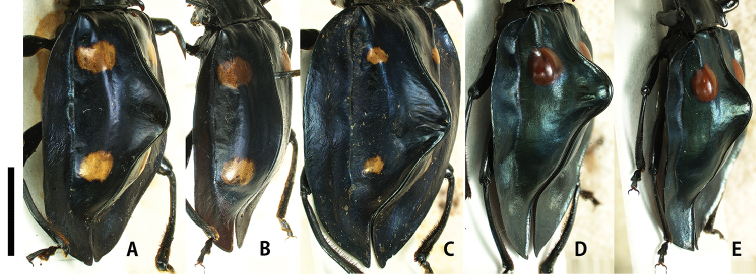
Elytra of *Eumorphus* spp. in dorso-lateral view. **A***E.costatus*, male **B***E.costatus*, female **C***E.wegneri*, male **D***E.mirabilis* sp. n., male **E***E.mirabilis* sp. n., female. Scale bar: 5.0 mm.

**Figure 4.**
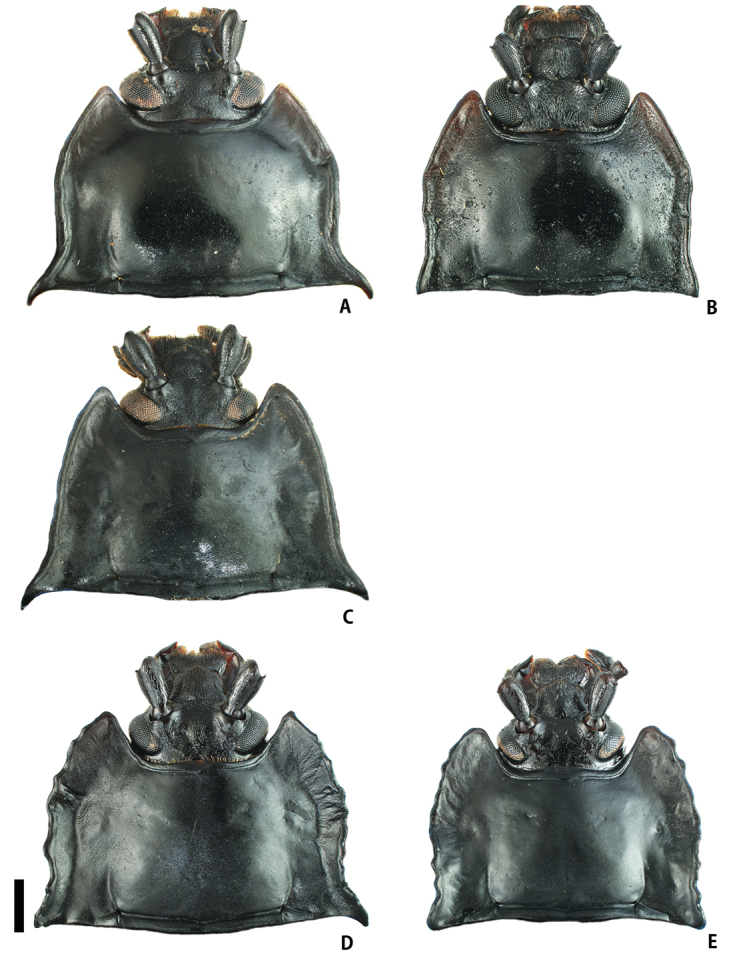
Head and pronotum of *Eumorphus* spp. **A***E.costatus*, male **B***E.costatus*, female **C***E.wegneri*, male **D***E.mirabilis* sp. n., male **E***E.mirabilis* sp. n., female. Scale bar: 1.0 mm.

**Figure 5.**
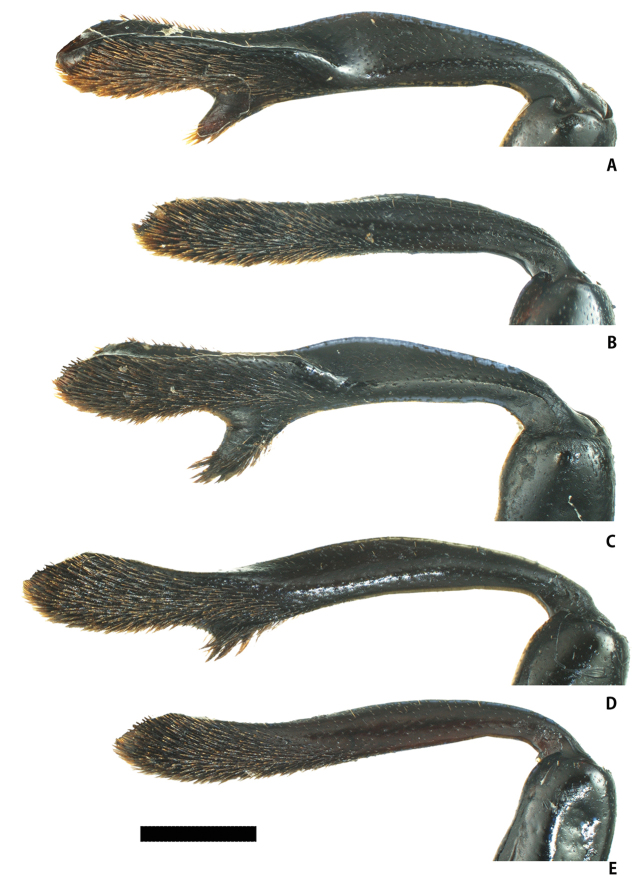
Fore tibia of *Eumorphus* spp. in dorsal view. **A***E.costatus*, male **B***E.costatus*, female **C***E.wegneri*, male **D***E.mirabilis* sp. n., male **E***E.mirabilis* sp. n., female. Scale bar: 1.0 mm.

**Figure 6.**
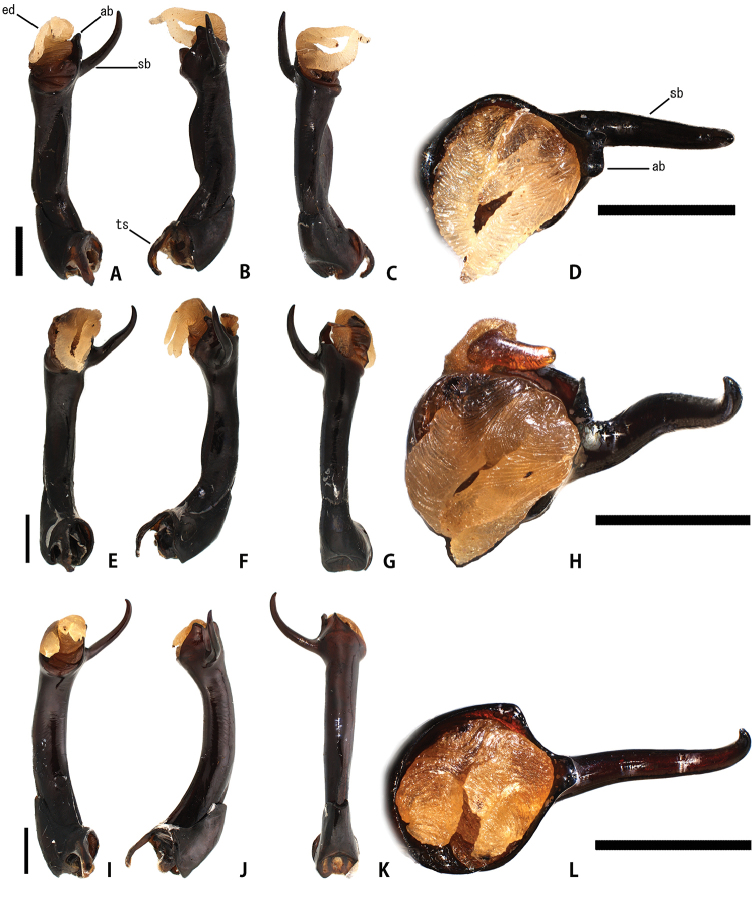
Aedeagus of *Eumorphus* spp. **A–D***E.costatus***E–H***E.wegneri***I–L***E.mirabilis* sp. n. **A, E, I** ventral **B, F, J** lateral **C, G, K** dorsal **D, H, I** apical. Abbreviations: ab: apical branch ed: endophallus sb: subapical branch ts: tegminal strut. Scale bars: 1.0 mm.

Figs 2A, B ; 3D, E ; 4D, E ; 5D, E ; 6I–L

#### Material examined.

Holotype, male (EUMJ), “(C. SULAWESI) Puncak Dingin, alt. ca. 1700 m, 14.XI.1985, S. Nagai leg.” Paratypes, female (EUMJ), same locality and collector, but 19.X.1985; 1 male and 1 female (NSMT), “Puncak Palopo, C. Celebes, IV.1989”.

#### Diagnosis.

This species can be easily separated from all other *Eumorphus* species in having cordiform elytra possessing one pair of spots and the pronotum laterally serrated.

#### Description.

*Male*.

Coloration of body black, with blueish luster; elytral spots reddish orange.

Antennae long, reaching ca. basal 1/4 of elytra; antennal club wide.

Pronotum (Fig. 4D) glossy, widely depressed in lateral parts; lateral margins irregularly serrate; lateral sulci relatively long, reaching ca. basal 1/3 of PLM; anterior angles nearly right-angled; posterior angles sharply pointed, projecting postero-ventrally; PLM/PLS 0.82–0.87 (0.84); PWP/PWA 1.84–1.99 (1.91); PWP/PLM 1.86–1.93 (1.90); PWP/PLS 1.53–1.68 (1.60). Elytra (Fig. 2A) cordiform, widest at anterior 1/4; lateral flattened margins wide, undulate vertically; elytral spots one pair, present in anterior 1/4, relatively large and oval, each distant from suture by half of its diameter; elytra along suture (Fig. 3D) distinctly elevated in basal 1/3; EL/EW 0.86–1.07 (0.97); EL/PLM 4.02–4.41 (4.21); EW/PWP 2.21–2.42 (2.31); TL/EW 1.07–1.31 (1.19). Fore tibiae (Fig. 5D) slender, slightly curved laterally in apical parts; apical carinae weakly projecting dorsally, covered with yellowish brown pubescence in dorsal and inner parts; fore tibial tooth short, situated in apical 1/4. Metatibial apices shortly prolonged in form of bifid projection; with outer angles shorter than inner ones.

Aedeagus (Fig. 6I–L) 5.4 mm long, relatively slender; median lobe gently curved ventrally; apical branch short, trapezoidal in apical part, with small projection in dorsal part of apical branch; subapical branch slender and long, evenly curved antero-laterally; tegmen movable, with relatively long tegminal strut; endophallus consisting of bifid long right membranous lobe and long left membranous lobe.

*Female*. Similar to male, but sexual dimorphism distinct in the following characteristics: posterior angles of pronotum (Fig. 4E) ca. 50°; fore tibiae (Fig. 5E) slender, slightly curved laterally in apical parts, covered with yellowish brown pubescence in apical half; metatibial apices simple; PLM/PLS 0.77–0.78 (0.78); PWP/PWA 1.60–1.68 (1.64); PWP/PLM 1.78–1.91 (1.85); PWP/PLS 1.37–1.50 (1.44); EL/EW0.99–1.07 (1.03); EL/PLM 4.45–4.46 (4.45); EW/PWP 2.33–2.35 (2.34); TL/EW 1.21–1.32 (1.26).

*Measurements*. Male (n = 2). TL 16.55–18.65 (17.60) mm; PW 6.37–6.43 (6.40) mm; PW 3.20–3.50 (3.35) mm; PL 3.30–3.45 (3.38) mm; PL 3.80–4.20 (4.00) mm; EL 13.25–15.20 (14.23) mm; EW 14.20–15.40 (14.80) mm. Female (n = 2). TL 16.23–16.38 (16.31) mm; PW 5.35–5.70 (5.53) mm; PW 3.35–3.40 (3.38) mm; PL 2.98–3.00 (2.99) mm; PL 3.80–3.90 (3.85) mm; EL 13.25–13.38 (13.32) mm; EW 12.45–13.40 (12.93) mm.

#### Distribution.

Indonesia (Sulawesi: Central).

## Supplementary Material

XML Treatment for Eumorphus

XML Treatment for Eumorphus costatus

XML Treatment for Eumorphus wegneri

XML Treatment for Eumorphus mirabilis
